# Entropy Based Modelling for Estimating Demographic Trends

**DOI:** 10.1371/journal.pone.0137324

**Published:** 2015-09-18

**Authors:** Guoqi Li, Daxuan Zhao, Yi Xu, Shyh-Hao Kuo, Hai-Yan Xu, Nan Hu, Guangshe Zhao, Christopher Monterola

**Affiliations:** 1 Department of Precision Instrument, Center for Brain-Inspired Computing Research, Tsinghua University, Beijing, P.R.China; 2 Institute of High Performance Computing, A*STAR, Singapore, Singapore; 3 School of Businesses, Renmin University of China, Beijing, P.R.China; 4 School of computing engineering, Nanyang Technological University, Singapore, Singapore; 5 School of Aerospace, Xi’an Jiaotong University, Xi’an, P. R. China; Tianjin University, CHINA

## Abstract

In this paper, an entropy-based method is proposed to forecast the demographical changes of countries. We formulate the estimation of future demographical profiles as a constrained optimization problem, anchored on the empirically validated assumption that the entropy of age distribution is increasing in time. The procedure of the proposed method involves three stages, namely: 1) Prediction of the age distribution of a country’s population based on an “age-structured population model”; 2) Estimation the age distribution of each individual household size with an entropy-based formulation based on an “individual household size model”; and 3) Estimation the number of each household size based on a “total household size model”. The last stage is achieved by projecting the age distribution of the country’s population (obtained in stage 1) onto the age distributions of individual household sizes (obtained in stage 2). The effectiveness of the proposed method is demonstrated by feeding real world data, and it is general and versatile enough to be extended to other time dependent demographic variables.

## Introduction

Predicting demographic trends (DT) [1] in the light of emerging complex processes [2] of the 21st Century continues to be an important and open research topic. Understanding developments and the changes in population is critical in assisting governments in targeting policies for the future and saving money for education, public health, retirement, transportation, energy consumption among others [3][4]. Specifically, DT refers to the changes in the joint distribution between population with time, age or other demographic factors, such as household’s size, health measures, economic status, religious affiliation, education, marriage, etc [5][6][7].

Forecasting DT is a challenging task, and remains to be a fundamental concern in both basic and applied ecology [8]. The complexity lies in the DT’S intricate connectivity to the heterogeneous activities of a large group of individuals, and it is impacted by observed and unobserved time dependent factors [9][10]. Existing methods such as the least square methods [11][12][13] and Bayesian inference [14], in spite of being the most extensively used procedures in estimating and predicting various engineering problems, fail to capture the driving mechanisms of complex processes that shapes DT [15]. There are very few literatures on building optimization models for understanding DT. Typical approaches involve incorporating factors such as environmental [16][17][18], demographic [19] and/or observer-related covariates [20]. However, data to support and verify such techniques is often not readily available as [21]–[23] suggesting that building an optimization model constrained by limited data to characterise DT is fundamentally important with a lot of potential applications.

Entropy-based methods, the measure of the uncertainty in random variables, have been successfully applied to many modelling and estimation problems, as seen in [24][25][26][27]. In this paper, we introduce the entropy-based method to estimate DT. We build the model motivated by our empirical observation that the age distribution of population follows an increasing entropy trend. The paradigm is based on minimizing the entropy-based objective function and incorporating some parameters describing the historical trends into the constraints where the dynamic and intrinsic properties can be reflected. We illustrate this procedure by estimating the evolution of demographic distributions over ages and household sizes. Our work involves a three-fold modeling stages. Firstly, an “age-structured population model” based on Leslie matrix [28][29][30][31] is used to predict the age distribution of a country’s population. This makes the modelling of the demographic temporal distributions become possible, as one usually needs to project the age distribution of population into other factors. Secondly, the age distribution of each household size is estimated based on a proposed entropy-based model, where we propose an entropy formulated cost function and incorporate the DT into the constraint conditions. The model applied in this stage is called “individual household size model”. Finally, the age distribution of the country’s population (obtained in stage 1) is projected onto the age distributions of individual household size types (obtained in stage 2), which we refer to as “total household size model”. Note that our estimation does not rely on any observed determinant on the formation of households. The evolution of the household size is estimated based on the historical information and the entropy principle.

To compare with existing works [3][32], our method predicts DT with limited information [33]. The output is a joint distribution of age and other demographic variables over time. Among its applications will be on policy analysis, economic forecasting and urban planning and so on. For the purpose of illustration, we use the population data from US Census and predict the age DT for each household size in 2010, based on the historical data in 2000 and 2006. The remaining parts of the paper are organized as follows. Section 2 lists the definitions and notations which are used throughout the article. Section 3 presents the three stages for the estimation of DT. The simulation results based US data are illustrated in Section 4 and we conclude the article in Section 5.

## Methodology

### Notations

In the following, we list the definitions and notations that will be used throughout the article:

*t*: The year index.
.^*T*^: Matrix transpose.
$\hat{.}$: The estimation of a variable.
*A*_*upper*_: The upper bound age.
*i*: The age index (*i* = 0, 1, …, *A*_*upper*_).
*P*_*i*_(*t*): The population for the people at age *i* (older than *i* but younger than *i* + 1) in the year *t*.
*P*(*t*) = [*P*_0_(*t*) … *P*_*i*_(*t*) … *P*_*A*_*upper*__(*t*)]^*T*^: The population vector for the people at all ages in the year *t*.
*N*_*i*_(*m*, *t*): The population for the male at age *i* in the year *t*.
*N*_*i*_(*f*, *t*): The population for the female at age *i* in the year *t*.
*N*(*m*, *t*) = [*N*_0_(*m*, *t*) *N*_1_(*m*, *t*) … *N*_*A*_*upper*__(*m*, *t*)]^*T*^: The population vector for the male at all ages.
*N*(*f*, *t*) = [*N*_0_(*f*, *t*) *N*_1_(*m*, *t*) … *N*_*A*_*upper*__(*f*, *t*)]^*T*^: The population vector for the female at all ages.
*D*_*i*_(*m*, *t*): The death rate for a male at age *i* in the year *t*.
*D*_*f*_(*i*, *t*): The death rate for a female at age *i* in the year *t*.
*B*_*i*_(*t*): The fertility rate for a female at age *i* in the year *t*.
*Ratio*_*mf*_(*t*): The ratio of the number of newly born boys to girls in the year *t*.
*Immig*(*m*, *t*): The male immigrants vector in the year *t*.
*Immig*(*f*, *t*): The female immigrants vector in the year *t*.
*Emig*(*m*, *t*): The male emigrants vector in the year *t*.
*Emig*(*f*, *t*): The female emigrants vector in the year *t*.
*m*_0_: Total number of household sizes.
*j*: The household size index (*j* = 1, 2, …, *m*_0_).
*k*_0_: Number of historical years’ data used in the individual household size Model (12).
*κ*: An index applied on the historical data for the year *t* − *κ* (*κ* = 0, 1, …, *k*_0_).
*G*_*n*_: The people in the age interval [0 *A*_*n*_] where *A*_*n*_ is an upper bound age of this group.
*n*: The group number index of *G*_*n*_ (*n* = 1, 2, …, *n*_0_) (as seen in Formula (10)).
*n*_0_: Number of groups (*G*_*n*_) in the individual household size Model (12).
$p_{i}^{j}\left(t\right)$: The probability (percentage) that people at age *i* in household size *j* in the year *t*.
*p*_*i*_(*t*) = [*p*_0_(*t*) … *p*_*A*_*upper*__(*t*)]^*T*^: Age distribution of the population in the year *t*.
$p^{j}\left(t\right)={\left[p_{0}^{j}\left(t\right)\;...\;p_{A_{upper}}^{j}\left(t\right)\right]}^{T}:$ Age distribution of household size *j* in the year *t*.
$q_{i}^{j}\left(t-\kappa \right)={\left[p_{0}^{j}\left(t-\kappa \right)\;...\;p_{A_{upper}}^{j}\left(t-\kappa \right)\right]}^{T}:$ Age distribution of household size *j* in the year *t* − *κ*.
*Entropy*(*t*): The entropy of the population distribution in the year *t*.
$\alpha_{n}^{j}\left(t+1\right)$: A ratio of people in group *G*_*n*_ to the population in household size *j* in Formula (10).
${\tilde{\alpha }}_{n}^{j}\left(t+1\right)$: A parameter defined in Model (12).
{.}_*i*_: The vector that contains the values of the variable {.} by changing subscript *i*.
$\omega_{0}^{*}$: A weight of the objective function in Model (12).
*ω*_1_, …, *ω*_*k*_0__: The weights defined in Model (12) and Formula (13).
$\xi_{k}^{j}\left(t+1\right):$ An error term in Formula (14).
$\bar{\xi }:$ The upper bound of *ξ*_*k*_(*t* + 1) and $\xi_{k}\left(t+1\right)\in \left[-\bar{\xi },\;\;\bar{\xi }\right]$.
*H*: The hessian matrix.
*x* ^*j*^(*t*): The number of household size *j* (*j* = 1, …, *m*_0_) in the year *t*.
$X\left(t\right)={\left[x^{1}\left(t\right)\;...\;x^{j}\left(t\right)\;...x^{m_{0}}\left(t\right)\right]}^{\prime }:$ The vector contains the number of each household size.
*W*: A weighting matrix in the total household size Model (16).
*τ*: A parameter in the matrix *W* in the total household size model (Formula (18)).
*u*: A small positive weight parameter in the total household size Model (16).
$\hat{F}:$ Predicted weighting matrix by collecting the predicted age distributions of all household sizes.

### Three stages for forecasting the demographic trends

**Fig 1:**
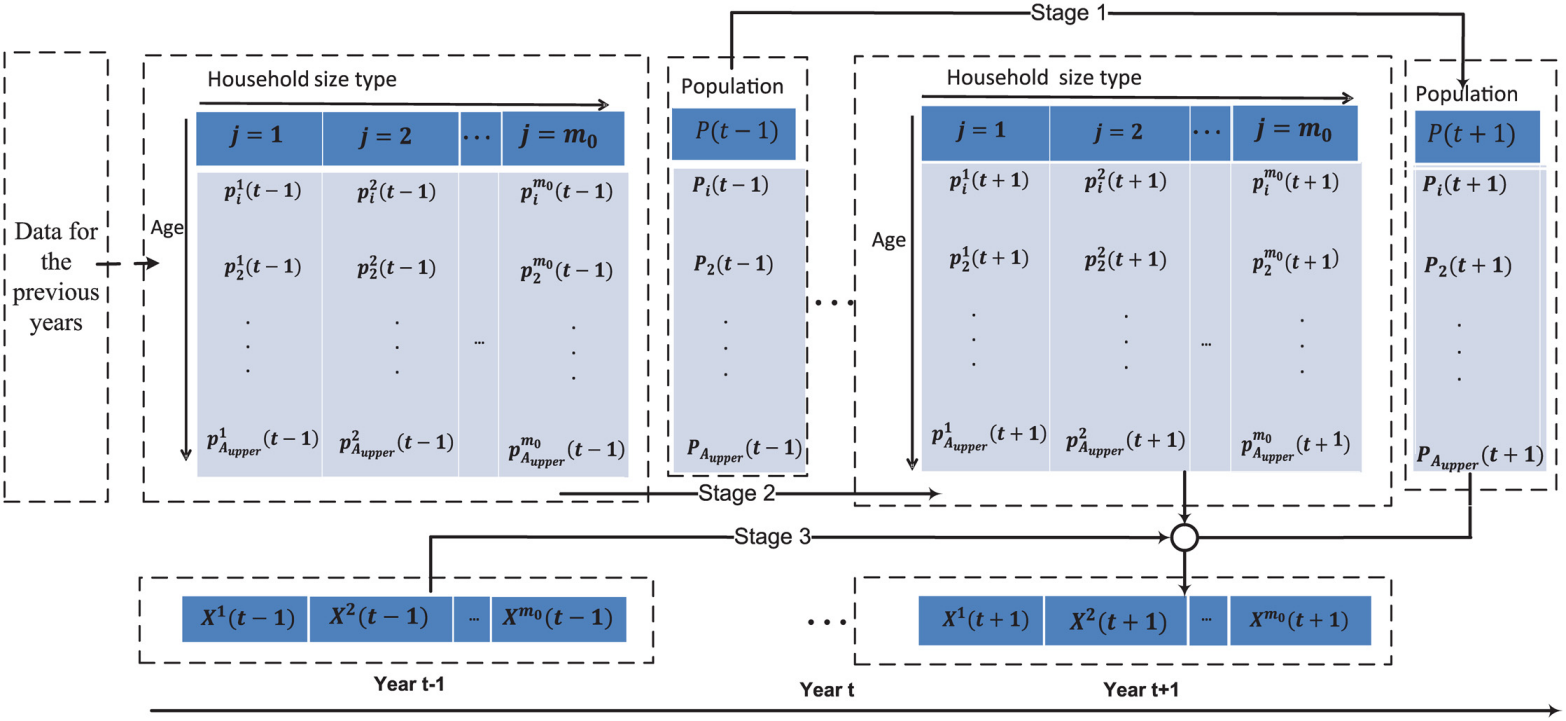
Illustration of the three stages for forecasting the demographic trends.

Fig 1 summarizes the three stages for forecasting the DT. Stage 1: using an “age-structured population model” to predict the population in the year *t* + 1. Stage 2: using an “individual household size model” to estimate the age distribution for each household size *j* based on data in the historical years where the DT reflected in the previous years can be incorporated into the constraint conditions. Stage 3: Combining the results from Stages 1 and 2, and employing a “total household size model” to predict the number of each household size. We detail in the next subsections each of the three stages shown.

### Age-structured population model: for estimating age distribution of the population

We consider the population as a summation of all the organisms of the same group or species, who live in the same geographical area, and have the capability of inter-breeding. Quite frequently, the prediction of demographic temporal distributions is highly linked to the population’s age-structure. Demographic temporal distribution modeling is achievable using the “age-structured population model” since it allows projection of the age distribution into other factors.

Assumptions. We apply the Leslie matrix method [28]–[31] that assumes:

- There is no plague, disaster or war that will lead to abrupt changes in age specific death rate.
- Statistical variables such as birth rates and birth ratio are slowly changed and predictable.
- The fertility rate for both local residents and immigrants is the same.
- All people who are older than *A*_*upper*_ are in the same age group. Here, we set *A*_*upper*_ = 90.

Problem formulation. We first consider the case without immigration and emigration. In the year *t* + 1, the number of people at age *i* + 1 is

$$\begin{array}{cc} P_{i+1}\left(t+1\right) & =N_{i+1}\left(m,t+1\right)+N_{i+1}\left(f,t+1\right)\\ & =\left[1-D_{i}\left(m,t\right)\right]N_{i}\left(m,t\right)+\;\left(1-D_{i}\left(f,t\right)\right)N_{i}\left(f,t\right) \end{array}$$ (1)

where *t* and *t* + 1 denote the current year and the next year, respectively, and *i* = 0, 1, …, *A*_*upper*_ − 1 is the age index. When *i* = *A*_*upper*_, we have

$$\begin{array}{cc} P_{i\geq A_{upper}}\left(t+1\right)= & \;\;\left[1-D_{A_{upper}-1}\left(m,t\right)\right]\;N_{A_{upper}-1}\left(m,t\right)+\left[1-D_{A_{upper}-1}\left(f,t\right)\right]\;N_{A_{upper}-1}\left(f,t\right)\\ & +\left[1-D_{i\geq A_{upper}}\left(m,t\right)\right]\;N_{i\geq A_{upper}}\left(m,t\right)+\left[1-D_{i\geq A_{upper}}\left(f,t\right)\right]\;N_{i\geq A_{upper}}\left(f,t\right) \end{array}$$ (2)

Let [*i*_1_, *i*_2_] be the age interval that a female has the ability to give birth. Then, *P*_0_(*t* + 1) = *N*_0_(*m*, *t* + 1) + *N*_0_(*f*, *t* + 1) and

$$\begin{array}{cc} N_{0}\left(m,t+1\right) & =\frac{Ratio_{mf}\left(t\right)}{1+Ratio_{mf}\left(t\right)}\sum_{i=i_{1}}^{i=i_{2}}B_{i}\left(t\right)\;N_{i}\left(f,t\right)\\ N_{0}\left(f,t+1\right) & =\frac{1}{1+Ratio_{mf}\left(t\right)}\sum_{i=i_{1}}^{i=i_{2}}B_{i}\left(t\right)\;N_{i}\left(f,t\right) \end{array}$$ (3)

where *Ratio*_*mf*_(*t*) is a ratio of the newly born boys (*N*_0_(*m*, *t*)) to the newly born girls (*N*_0_(*f*, *t*)) at year *t*. Let

$$\begin{array}{cc} P(t) & ={\left[P_{0}\left(t\right)\;P_{1}\left(t\right)\;...\;P_{A_{upper}}\left(t\right)\right]}^{T}\\ N(m,t) & ={\left[N_{0}\left(m,t\right)\;N_{1}\left(m,t\right)\;...\;N_{A_{upper}}\left(m,t\right)\right]}^{T}\\ N(f,t) & ={\left[N_{0}\left(f,t\right)\;N_{1}\left(f,t\right)\;...\;N_{A_{upper}}\left(f,t\right)\right]}^{T} \end{array}$$ (4)

be the vectors of the population, male population and female population, respectively, for ages between 0 and *A*_*upper*_ at year *t*.

Next, we extend the model to take into account of immigration effects. Let *Immig*(*m*, *t*)/*Emig*(*m*, *t*) and *Immig*(*f*, *t*)/*Emig*(*f*, *t*) be the respective immigrants and emigrants vector for males/females at year *t*. We obtain the “age-structured population model” as follows:

$$\begin{array}{cc} P(t+1) & =N(m,t+1)+N(f,t+1)\\ N(m,t+1) & =A\left(t\right)N\left(m,t\right)+\frac{Ratio_{mf}\left(t\right)}{1+Ratio_{mf}\left(t\right)}B\left(t\right)N\left(f,t\right)\\ & \;\;+Immig(m,t)-Emig(m,t)\\ N(f,t+1) & =[C\left(t\right)+\frac{1}{1+Ratio_{mf}\left(t\right)}B\left(t\right)]N\left(f,t\right)\\ & \;\;+Immig(f,t)-Emig(f,t) \end{array}$$ (5)

where *A*(*t*), *B*(*t*) and *C*(*t*) are the matrices constructed based on Eqs (2)–(4), and given by

$$A(t)=\left[\begin{array}{ccccccc} 0 & 0 & 0 & \ldots & 0 & 0 & 0\\ 1-D_{0}(m,t) & 0 & 0 & \ldots & 0 & 0 & 0\\ 0 & 1-D_{1}(m,t) & 0 & \ldots & 0 & 0 & 0\\ \ldots & \ldots & \ldots & \ldots & \ldots & \ldots & \ldots \\ 0 & 0 & 0 & \ldots & 0 & 0 & 0\\ 0 & 0 & 0 & \ldots & 0 & 1-D_{A_{upper}-1}(m,t) & 1-D_{i\geq A_{upper}}(m,t) \end{array}\right]$$ (6)

$$B(t)=\left[\begin{array}{ccccccccc} 0 & \ldots & 0 & B_{i_{1}}(t) & \ldots & B_{i_{2}}(t) & 0 & \ldots & 0\\ 0 & \ldots & 0 & 0 & \ldots & 0 & 0 & \ldots & 0\\ \ldots & \ldots & \ldots & \ldots & \ldots & \ldots & \ldots & \ldots & \\ 0 & \ldots & 0 & 0 & \ldots & 0 & 0 & \ldots & 0 \end{array}\right]$$ (7)

$$C(t)=\left[\begin{array}{cccccccc} 0 & 0 & \ldots & 0 & \ldots & 0 & \ldots & 0\\ 1-D_{0}(f,t) & 0 & \ldots & 0 & \ldots & 0 & \ldots & 0\\ 0 & 1-D_{1}(f,t) & \ldots & 0 & \ldots & 0 & \ldots & 0\\ \ldots & \ldots & \ldots & \ldots & \ldots & \ldots & \ldots & \ldots \\ 0 & 0 & \ldots & 0 & \ldots & 0 & 1-D_{A_{upper}-1}(f,t) & 1-D_{i\geq A_{upper}}(f,t) \end{array}\right]$$ (8)

Note that the population data we collected allows us to estimate the values of all the above parameters (such as the fertility rates and death rates). These parameters change slowly and are predictable which confirm the validity of our assumption. Thus, the population distribution for the coming years can be predicted based on the age-structured population Model (5), and its estimation is denoted as $\hat{P}\left(t+1\right)$ for the year *t* + 1 as shown in Model (16) later.

### Individual household size model: for estimating age distribution for each household size

In this section, we will describe in detail our *individual household size model* that estimates the age distribution of each household size. The model is operated by minimizing an entropy based objective function and using the historical trends as constraints, where both the dynamic and intrinsic properties are reflected.

Let *p*_*i*_(*t*) be the probability that a person is at age *i* in year *t*. We define an entropy function for year *t* as follows:

$$\begin{array}{c} Entropy\left(t\right)=-\sum_{i=0}^{A_{upper}}p_{i}\left(t\right)ln\left(p_{i}\left(t\right)\right) \end{array}$$ (9)

where $\Sigma_{i=0}^{A_{upper}}p_{i}\left(t\right)=1$ and *p_i_*(*t*) ≥ 0.

**Fig 2:**
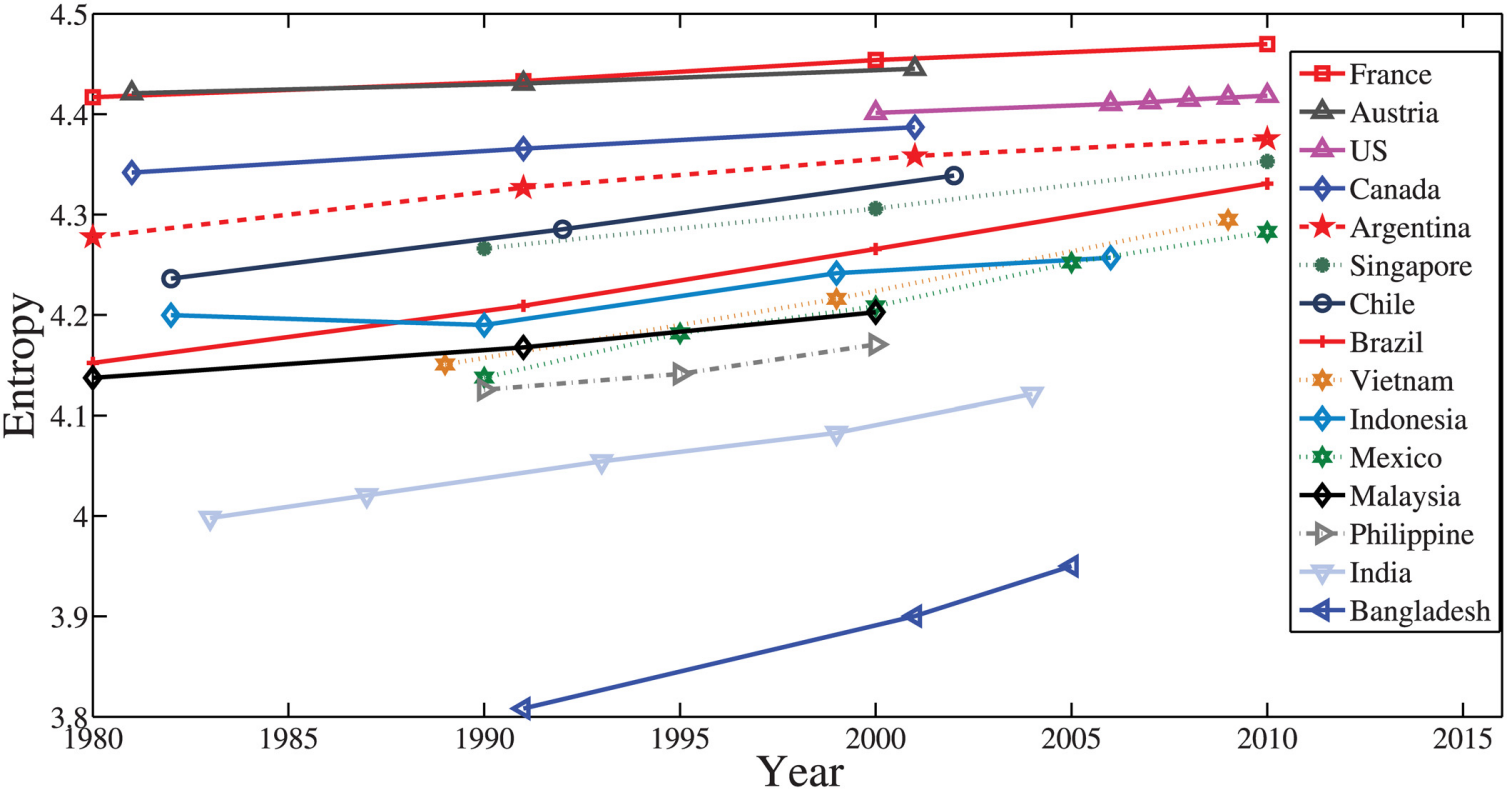
The age distribution entropy of selected countries as a function of time. Note that there is a slight decrease in the entropy of the Indonesia’s population from 1990 to 2000 perhaps due to the difference in the statistics method used in the years (1990 vs 2000) considered as indicated in https://international.ipums.org/international/.

Fig 2 plots the entropy of the age distribution based on the population data collected from six countries. In general, the entropy of the age distribution increases monotonically with respect to time in most countries. This observation suggests that we can estimate the age distribution of a particular household size based on entropy concepts. To this end, we divide the household size into *n*_0_ types: i.e., 1 person per household, 2 persons per household, …, until *n*_0_ persons per household.

Let *j* be the household size index and assume that we already have the age distributions for each household size *j* (*j* ∈ {1, …, *m*_0_}) in the years *t*, *t* − 1, …, *t* − *k*_0_, which are denoted as $q_{i}^{j}\left(t-\kappa \right)$ for *κ* = 0,1, …, *k*_0_. Let $p_{i}^{j}\left(t+1\right)$ represent the percentage of persons at age *i* in household size *j* in the year *t* + 1. This means we group the people whose ages are above 90 years together. Our objective is to estimate the age distribution $p_{i}^{j}\left(t+1\right)$ in the year *t* + 1 based on the historical data.

We group the people from 0 to *A*_*upper*_ years old into *n*_0_ groups, i.e., the groups *G*_*n*_ for *n* = 1, …, *n*_0_, where *n*_0_ ≪ *A*_*upper*_. The age interval for the group *G*_*n*_ is [0, *A*_*n*_] and 0 < *A*_1_ < *A*_2_ < … < *A*_*n*_0__ = *A*_*upper*_. It is easy to see that *G*_*n*−1_ ⊂ *G*_*n*_. Define $\alpha_{n}^{j}(t)$ as a parameter such that

$$\begin{array}{c} \alpha_{n}^{j}\left(t\right)=\sum_{i=0}^{A_{n}}p_{i}^{j}\left(t\right) \end{array}$$ (10)

which means that $\alpha_{n}^{j}(t)$ is a ratio of people in group *G*_*n*_, i.e., in the age interval [0, *A*_*n*_], to the population in household size *j*. Note that ∀*j* ∈ {1, …, *m*_0_}, *α*_*n*_0__(*t* + 1) = 1 since *A*_*n*_0__ = *A*_*upper*_.

Let

$$\begin{array}{c} {\tilde{\alpha }}_{n}^{j}\left(t+1\right)=\alpha_{n}^{j}\left(t+1\right)-\alpha_{n}^{j}\left(t\right) \end{array}$$ (11)

be the parameter which reflects the percentage change of the ratio $\alpha_{n}^{j}(t)$ from the year *t* to the next year *t* + 1.

From here, we build an individual household size model to predict the age distribution ${\left\{p_{i}^{j}\left(t+1\right)\right\}}_{i}$ for each household size type *j* where *j* = 1, 2, …, *m*_0_, by optimizing the following:

$$\begin{array}{cc} min & \sum_{\kappa =0}^{k_{0}}\omega_{\kappa +1}\sum_{i=1}^{A_{upper}}p_{i}^{j}\left(t+1\right)ln\left(\frac{p_{i}^{j}\left(t+1\right)}{q_{i}^{j}\left(t-\kappa \right)}\right)\\ & +\omega_{0}^{*}\sum_{i=1}^{A_{upper}}p_{i}^{j}\left(t+1\right)ln\left(\frac{p_{i}^{j}\left(t+1\right)}{1/A_{upper}}\right)\\ s.t & \sum_{i=0}^{A_{1}}p_{i}^{j}\left(t+1\right)\leq \sum_{i=1}^{A_{1}}q_{i}^{j}\left(t\right)+{\tilde{\alpha }}_{1}^{j}\left(t+1\right)\\ & \;\;\;\;\;\;\;\;\;\;\;\;\;\;\;\;\vdots \\ & \sum_{i=0}^{A_{n}}p_{i}^{j}\left(t+1\right)\leq \sum_{i=1}^{A_{n}}q_{i}^{j}\left(t\right)+{\tilde{\alpha }}_{n}^{j}\left(t+1\right)\\ & \;\;\;\;\;\;\;\;\;\;\;\;\;\;\;\;\vdots \\ & \sum_{i=0}^{A_{n_{0}}}p_{i}^{j}\left(t+1\right)\leq \sum_{i=1}^{A_{n_{0}}}q_{i}^{j}\left(t\right)+{\tilde{\alpha }}_{n_{0}}^{j}\left(t+1\right)\\ & \sum_{\kappa =1}^{k_{0}+1}\omega_{\kappa }=1\\ & -p_{i}^{j}\left(t+1\right)\leq 0\;\;for\;\;i=0,1,....,A_{upper} \end{array}$$ (12)

Again, given that the entropy of the population is monotonically increasing with time, we can minimize an entropy based cost function under some constraints by employing the historical data. Compared with Eq (9), we omit the minus sign “−” such that the model becomes a minimization problem. The upper limit of such entropy as *t* = +∞ is a uniform distribution with a histogram function having a constant 1/*A*_*upper*_ magnitude. Essentially, there are two parts in this cost function where $\omega_{0}^{*}$ is a small positive weight parameter. The first part is the cross entropy distance (KL distance [34]) between ${\left\{p_{i}^{j}\left(t+1\right)\right\}}_{i}$ and the historical data, and the second part is the relative entropy distance between ${\left\{p_{i}^{j}\left(t+1\right)\right\}}_{i}$ and population distribution when *t* = +∞.

Note that we can never know the value of ${\tilde{\alpha }}_{n}^{j}(t+1)$ at the year *t* as we do not know $\alpha_{n}^{j}\left(t+1\right)$. However, it can be estimated from the historical data as:

$$\begin{array}{cc} {\hat{\tilde{\alpha }}}_{n}^{j}\left(t+1\right) & =\sum_{\kappa =1}^{k_{0}}\omega_{\kappa }\left(\alpha_{n}^{j}\left(t-\kappa \right)-\alpha_{n}^{j}\left(t\right)\right)/\kappa \\ & =\sum_{\kappa =1}^{k_{0}}\omega_{\kappa }\left(\sum_{i=0}^{A_{n}}q_{i}^{j}\left(t-\kappa \right)-\sum_{i=0}^{A_{n}}q_{i}^{j}\left(t\right)\right)/\kappa \end{array}$$ (13)

where *ω*_*κ*_ for *κ* = 1, …, *k*_0_ + 1 are decreasing weights, which implies that the more recent data is more valued. Let $\xi_{n}^{j}\left(t+1\right)=\frac{{\tilde{\alpha }}_{n}^{j}\left(t+1\right)-{\hat{\tilde{\alpha }}}_{n}^{j}\left(t+1\right)}{{\hat{\tilde{\alpha }}}_{n}^{j}\left(t+1\right)}$ be an error term of the estimation, then we have

$$\begin{array}{c} {\tilde{\alpha }}_{n}^{j}\left(t+1\right)={\hat{\tilde{\alpha }}}_{n}^{j}\left(t+1\right)\left(1+\xi_{n}\left(t+1\right)\right) \end{array}$$ (14)

the distribution of $\xi_{n}^{j}\left(t+1\right)$ is known and bounded within $\left[-\bar{\xi },\bar{\xi }\right]$. Usually $\xi_{n}^{j}\left(t+1\right)$ can be assumed as a random variable uniformly distributed in $\left[-\bar{\xi },\bar{\xi }\right]$. We now have that:

Theorem 1. The optimization problem defined in Model (12) is a strict convex optimization.

Proof. Note that the Hessian matrix *H* of the objective function is given by:

$$\begin{array}{c} H=[\begin{array}{ccccc} \frac{\sum_{\kappa =0}^{k_{0}}\omega_{\kappa +1}}{p_{1}^{j}\left(t+1\right)} & 0 & 0 & ... & 0\\ 0 & \frac{\sum_{\kappa =0}^{k_{0}}\omega_{\kappa +1}}{p_{2}^{j}\left(t+1\right)} & 0 & ... & 0\\ & \vdots \vdots & \vdots & \ddots & \vdots \\ 0 & 0 & 0 & ... & \frac{\sum_{\kappa =0}^{k_{0}}\omega_{\kappa +1}}{p_{A_{upper}}^{j}\left(t+1\right)} \end{array}] \end{array}$$ (15)

Since $p_{i}^{j}\left(t+1\right)\geq 0$ for all *i* and *j*, it is easy to see that *H* is a positive definite matrix. On the other side, it is known that the constraints of the optimization problem in the Model (12) are linear. Therefore, the feasible domain is a convex set. Both the objective function and the feasible domain are convex, hence the problem is a convex optimization. Note that one only needs to find a local minimum point of a convex optimization to obtain the global minimum point [35][36][37][38].

### Total household size model: for estimating the number of each household size

In this section, we build a *total household size model* to further estimate the number of each household size *j* for *j* = 1, 2, …, *m*_0_ based on the predicted age distribution of population and age distribution of each individual household size. Here, our objective is to estimate the number of household size *j* for *j* = 1, 2, …, *m*_0_ in the year *t* + 1.

Let *x* ^*j*^(*t*) be the number of household with size *j* in the year *t* and denote that $X\left(t\right)={\left[x^{1}\left(t\right)\;...\;x^{m_{0}}\left(t\right)\right]}^{T}$. We hope to estimate the vector $X\left(t+1\right)={\left[x^{1}\left(t+1\right)\;...\;x^{m_{0}}\left(t+1\right)\right]}^{T}$. As mentioned, the first stage is to obtain the estimated total population distribution $\hat{P}\left(t+1\right)$ based on the current fertility rate and death rate. The second stage is then to obtain the estimated age distribution of each household type *j* denoted as ${\hat{p}}^{j}\left(t+1\right)$. Now we estimate the household number distribution by solving the following total household size model:

$$\begin{array}{cc} min & \left(1-u\right)\cdot \left|\right|\hat{F}\cdot X\left(t+1\right)-\hat{P}{\left(t+1\right))||}^{2}+u\cdot ||W\cdot X\left(t+1\right)-{X\left(t\right))||}^{2}\\ s.t & X(t+1)>0 \end{array}$$ (16)

where ∣∣.∣∣ is the *L*_2_ norm, and *X*(*t* + 1) ≥ 0 means each component of *X*(*t* + 1) is nonnegative, and $\hat{F}$ is a weighting matrix collected from the the predicted age distributions of all household sizes:

$$\hat{F}=\left[\begin{array}{cccc} 1\cdot {\hat{p}}_{1}^{1}(t+1) & 1\cdot {\hat{p}}_{2}^{1}(t+1) & \ldots & 1\cdot {\hat{p}}_{A_{upper}}^{j}(t+1)\\ 2\cdot {\hat{p}}_{1}^{2}(t+1) & 2\cdot {\hat{p}}_{2}^{2}(t+1) & \ldots & 2\cdot {\hat{p}}_{A_{upper}}^{2}(t+1)\\ \vdots & \vdots & \ldots & \vdots \\ m_{0}\cdot {\hat{p}}_{1}^{m_{0}}(t+1) & m_{0}\cdot {\hat{p}}_{2}^{m_{0}}(t+1) & \ldots & 1\cdot {\hat{p}}_{A_{upper}}^{m_{0}}(t+1) \end{array}\right]$$ (17)

and *W* is a diagonal weighting matrix for different household size type given by

$$W=\left[\begin{array}{cccc} 1 & 0 & \ldots & 0\\ 0 & 2^{\tau } & \ldots & \vdots \\ \vdots & \vdots & j^{\tau } & \vdots \\ 0 & 0 & \ldots & m_{0}^{\tau } \end{array}\right]$$ (18)

The above objective function contains two parts with *u* being a small positive weight parameter. The first part is the distance between the estimated age distribution for population and the accumulative of the age distribution for all household sizes. The other part is the weighted distance of the estimated *X*(*t* + 1) (denoted as $\hat{X}\left(t+1\right)$) to *X*(*t*). As there are *j* persons in the household size *j*, we construct a diagonal weighting matrix *W* with a given power *τ* > 0 in Eq 18. As shown in Theorem 2, the optimization of Eq (16) is also convex.

Theorem 2. The optimization problem defined in Model (16) is convex.

Prof. The proof is similar to Theorem 1. The Hessian matrix of the objective function in Eq (16) is

$$\begin{array}{c} H=\left(1-u\right){\hat{F}}^{T}\;\hat{F}+uW^{T}W \end{array}$$ (19)

Obviously *H* is a positive definite matrix and we have this theorem holds.

## Simulations

In this section, we illustrate the procedure we have discussed above using the US’s Census population data. We predict the demographic distribution in the year 2010 based on the historical data in years 2000 and 2006. The prediction is then compared with the actual Census data in the year 2010. We show that the method we described here accurately captures the actual statistics. As mentioned, there are three stages in the estimation:

Stage 1: Estimating the age distribution of the population by employing the *age structure based population model* in Section 3A.
Stage 2: Estimating age distribution for each household size type by employing the *individual household size model* in Section 3B.
Stage 3: Estimating the number of different household size type by employing the *total household size model* in Section 3C.

**Fig 3:**
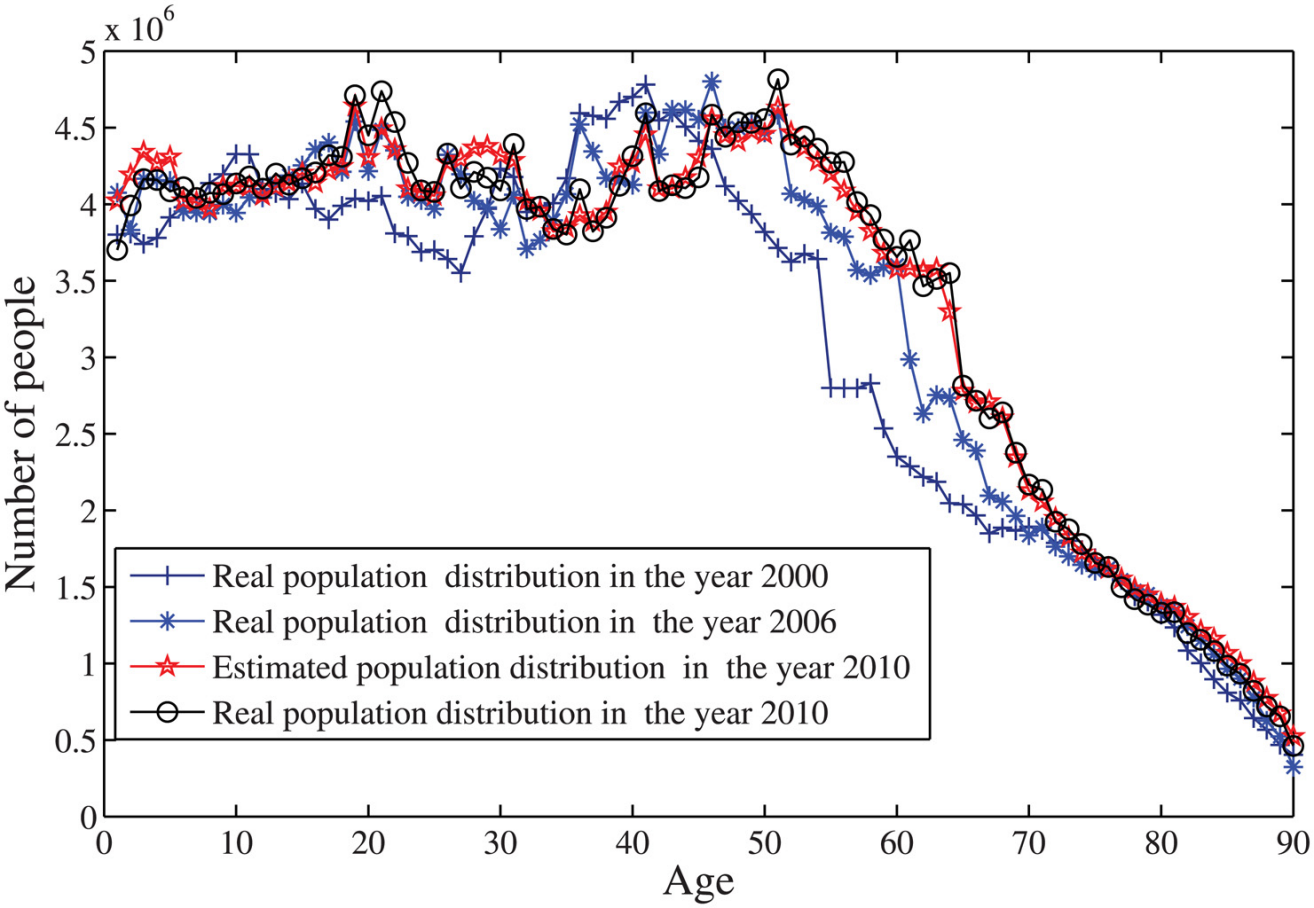
The predicted age distribution of US population for the year 2010 based on the population data in the years 2000 and 2006.

In Stage 1, we collect the population data from the US Census and get the values of all parameters that are required in Model (5). By solving this model, we obtain the estimation of the population in the year 2010 based on the the data in the year 2000 and 2006 in Fig 3.

**Fig 4:**
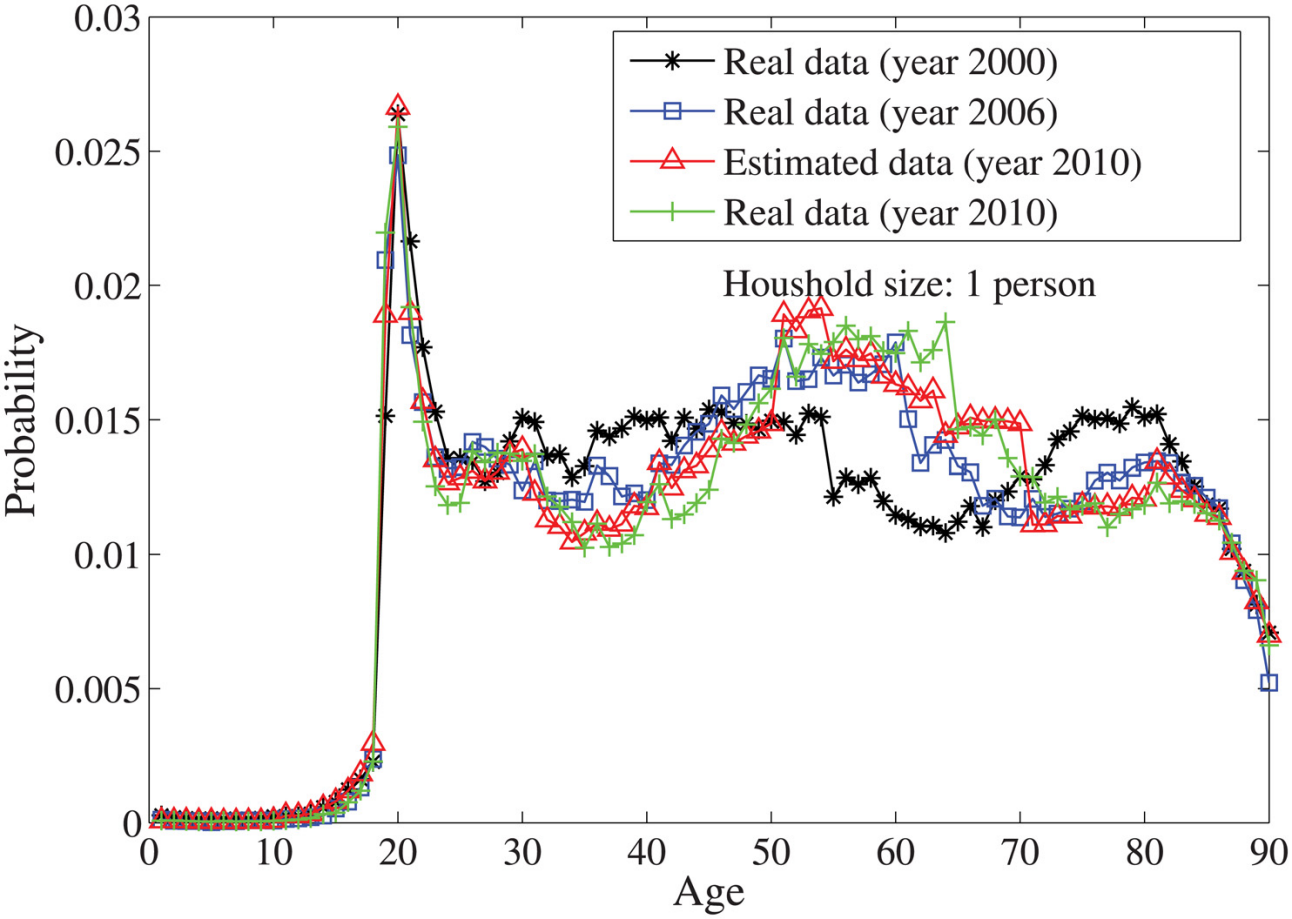
The estimated age distribution for household size type 1 in the year 2010 (x axis is the age index and y axis is the probability).

**Fig 5:**
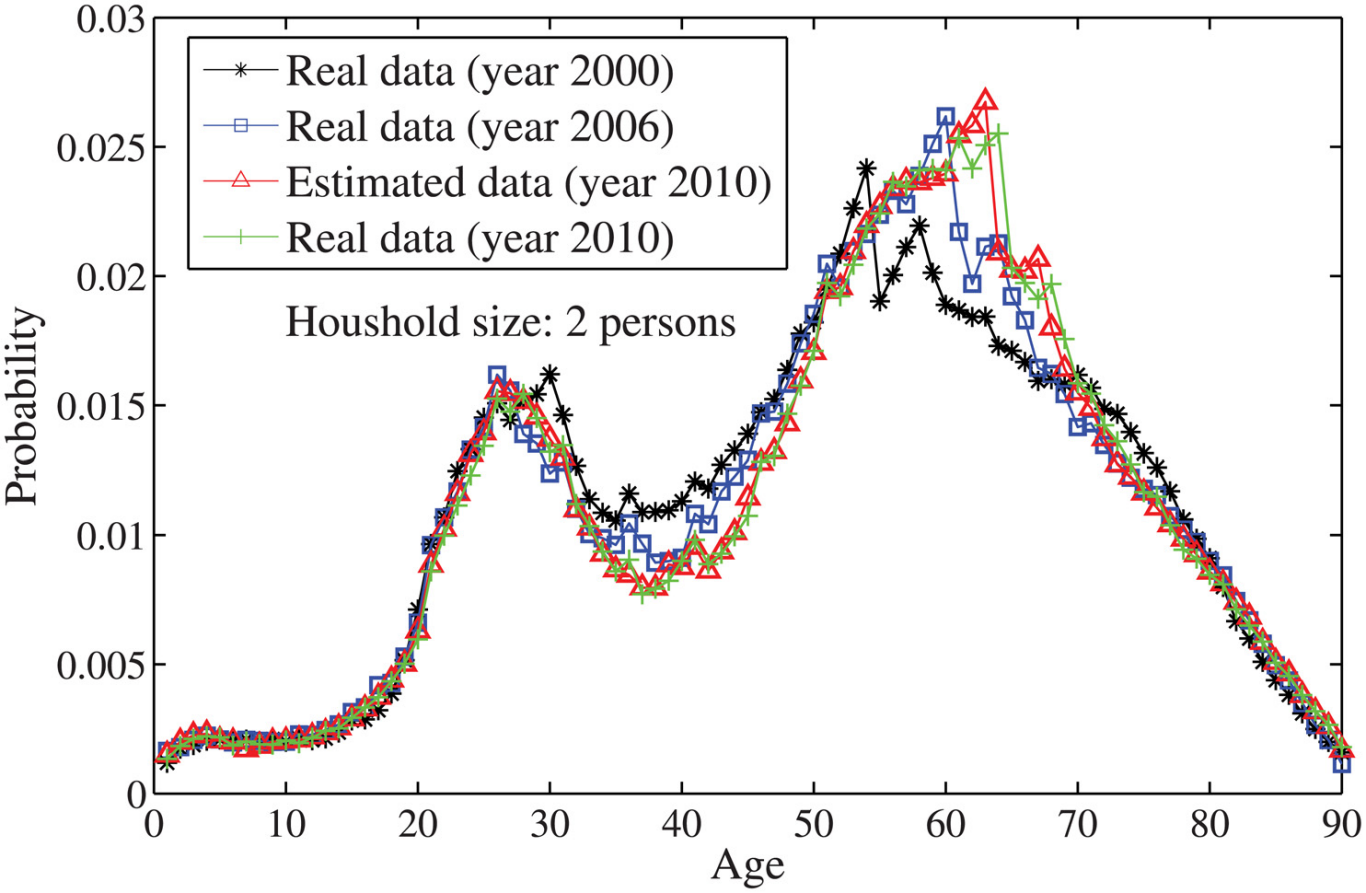
The estimated age distributions for household size 2 in the year 2010 using US data.

**Fig 6:**
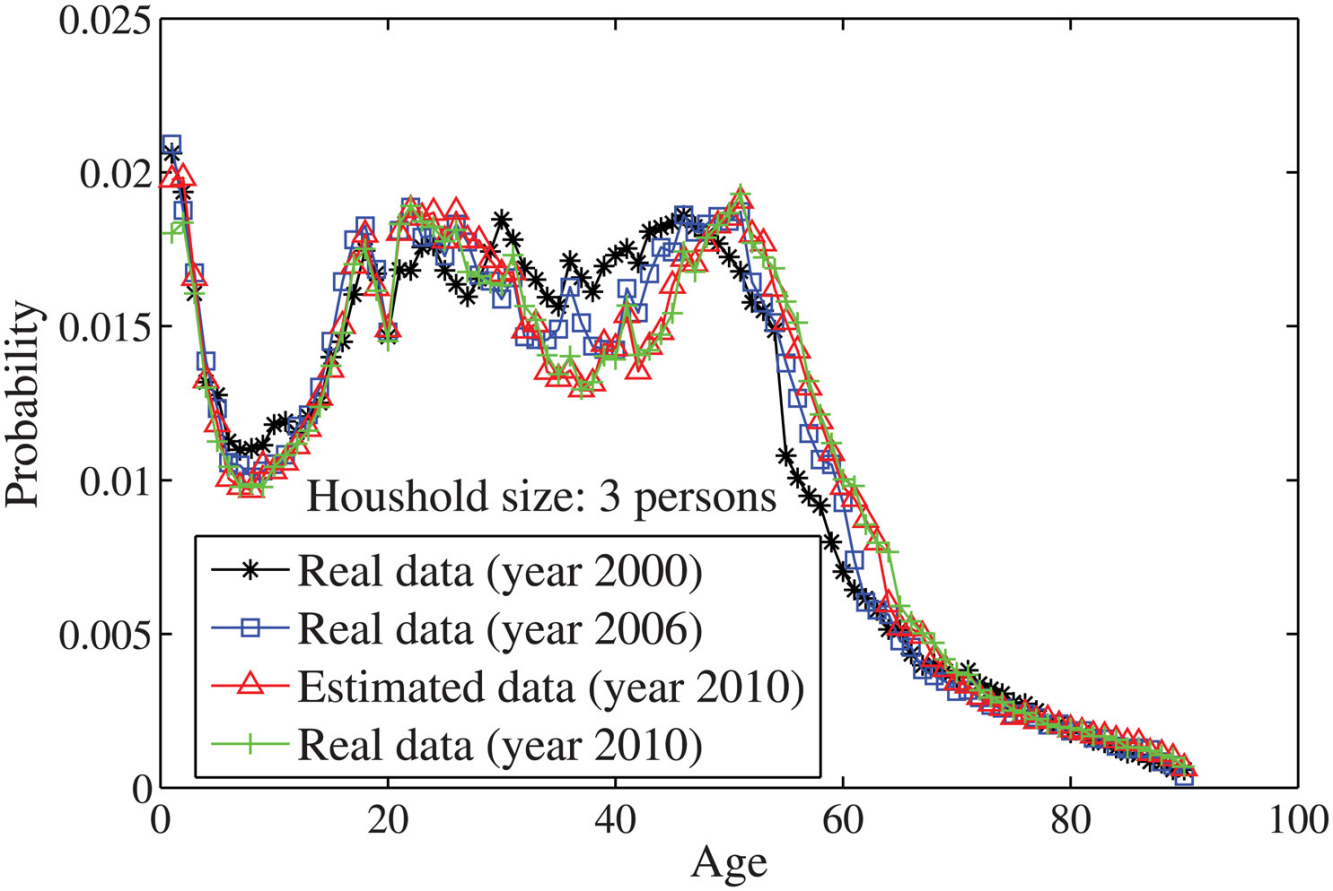
The estimated age distributions for household size 3 in the year 2010 using US data.

**Fig 7:**
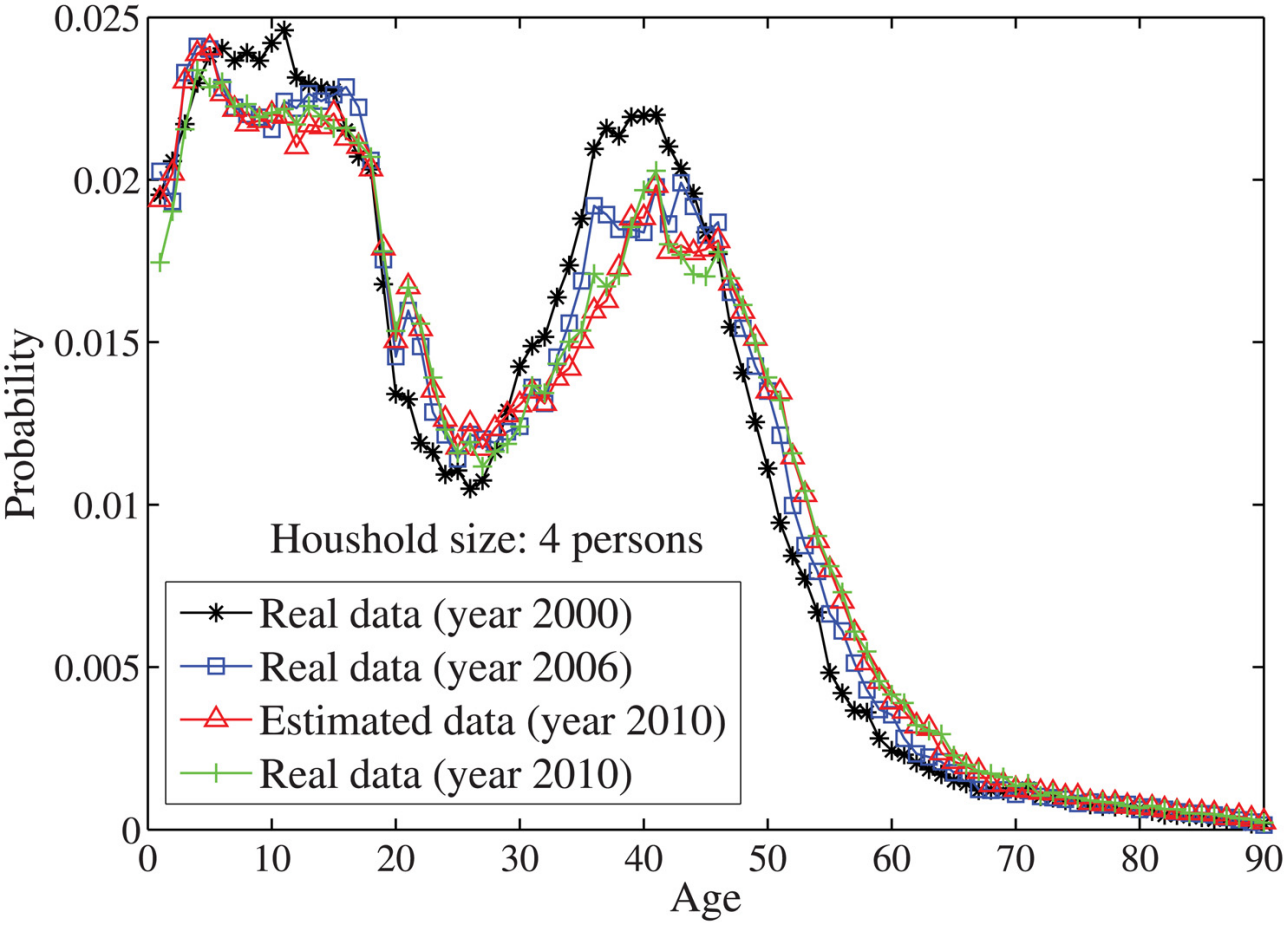
The estimated age distributions for household size 4 in the year 2010 using US data.

**Fig 8:**
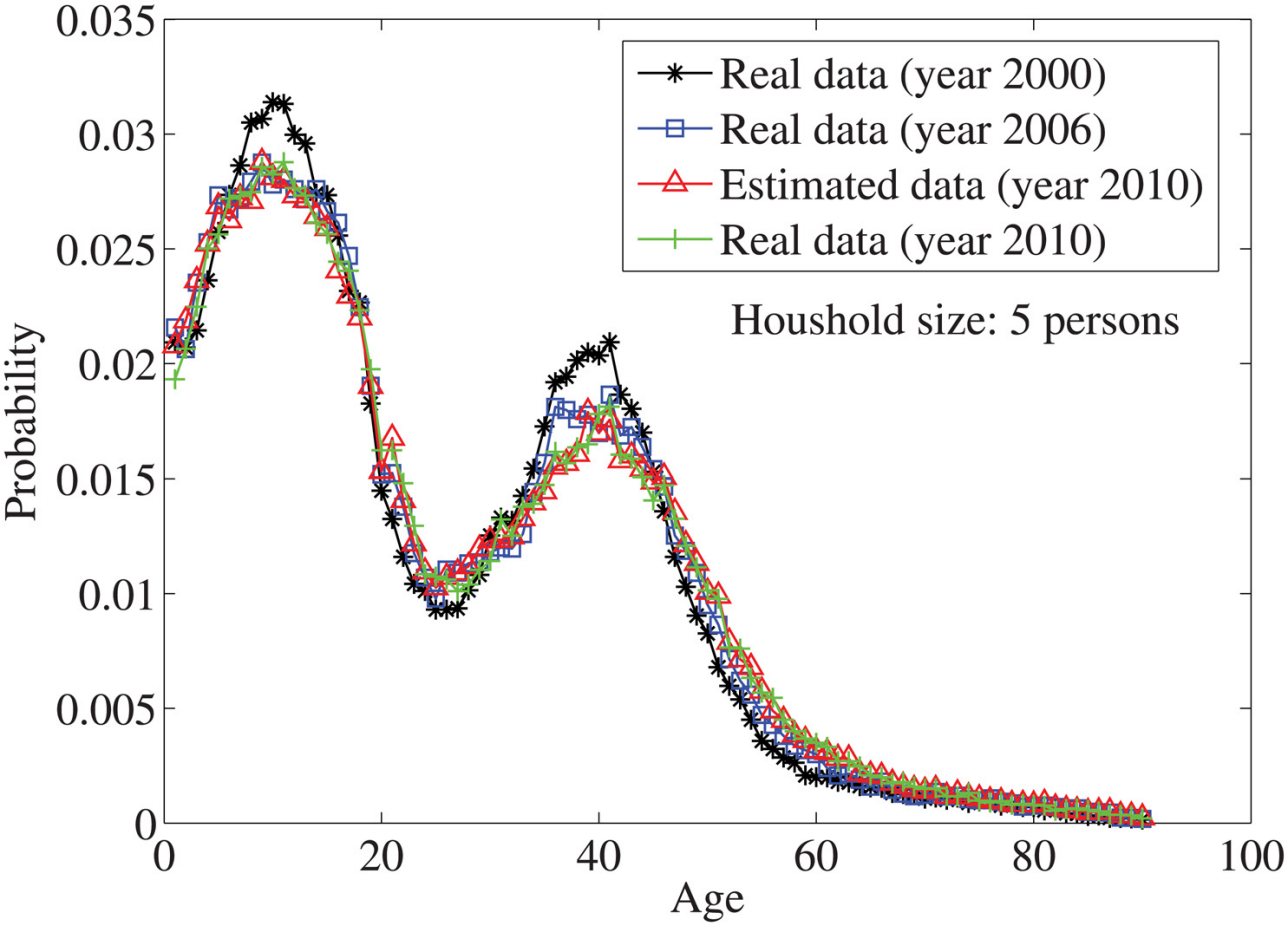
The estimated age distributions for household size 5 in the year 2010 using US data.

**Fig 9:**
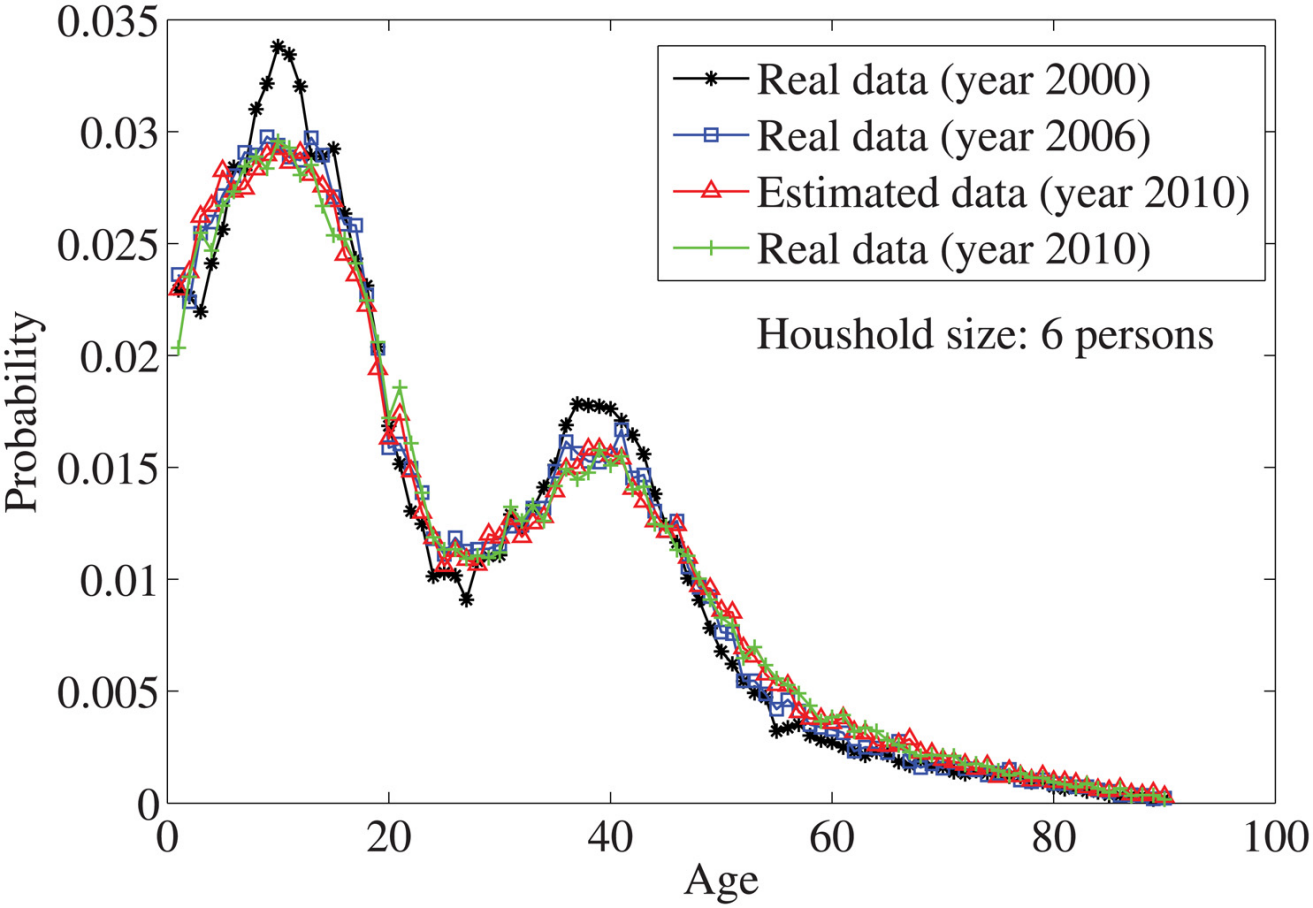
The estimated age distributions for household size 6 in the year 2010 using US data.

**Fig 10:**
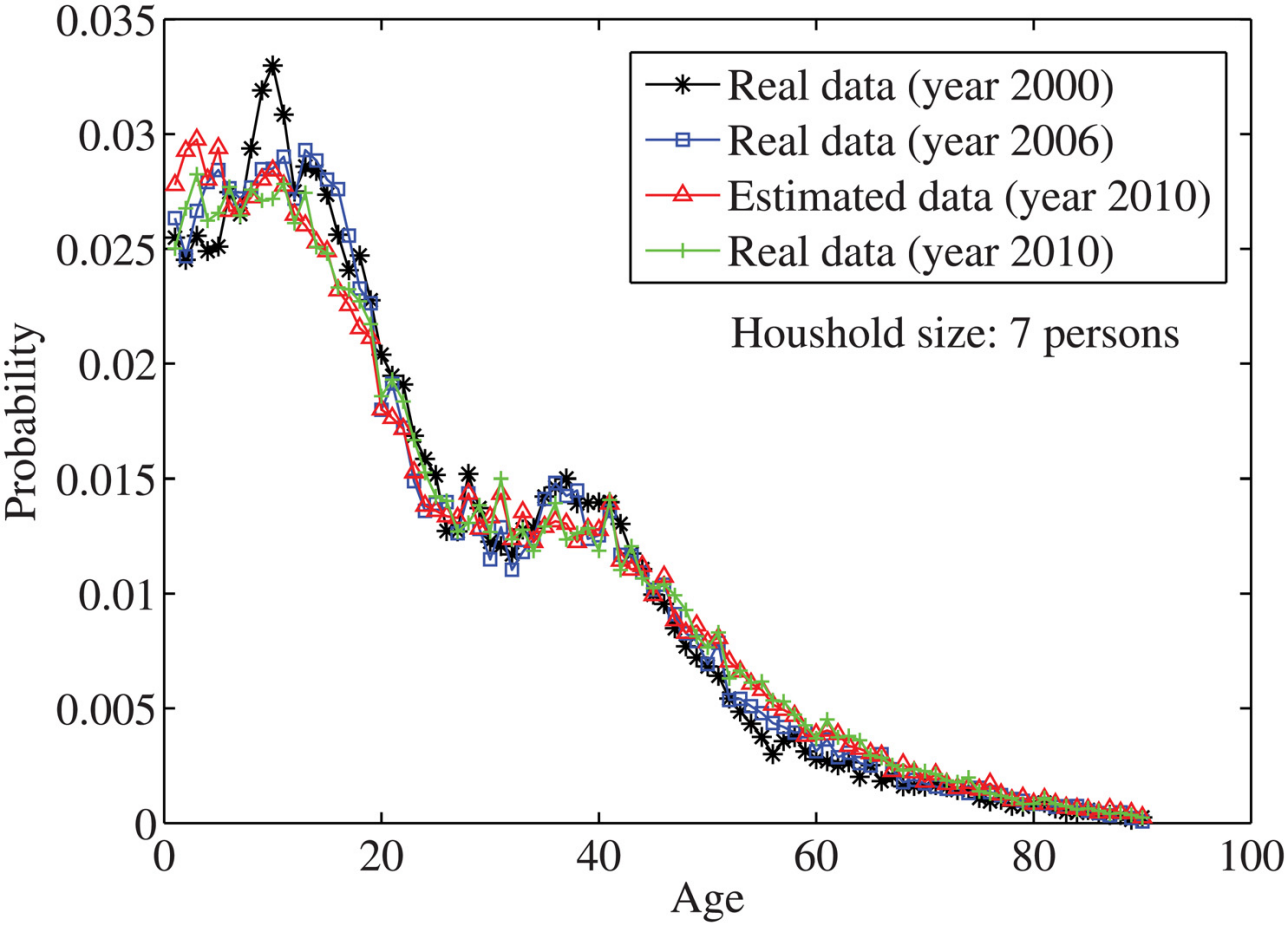
The estimated age distributions for household size 7 in the year 2010 using US data.

In Stage 2, by letting *ω* = [0.95 0.025 0.025] and assuming that the error term bound $\bar{\xi }=0$, we divide the population into 9 groups (*G*_*n*_, *n* = 1, 2, …, 9) and let *A*_*n*_ = *n*⋅10. By solving the individual household size model (12), the age distributions for the household sizes *j* = 1 and *j* = 2, …, 7 are obtained in Figs 4–10, respectively. It is seen that the individual household size model predicts accurately the age distribution of all household sizes.

**Fig 11:**
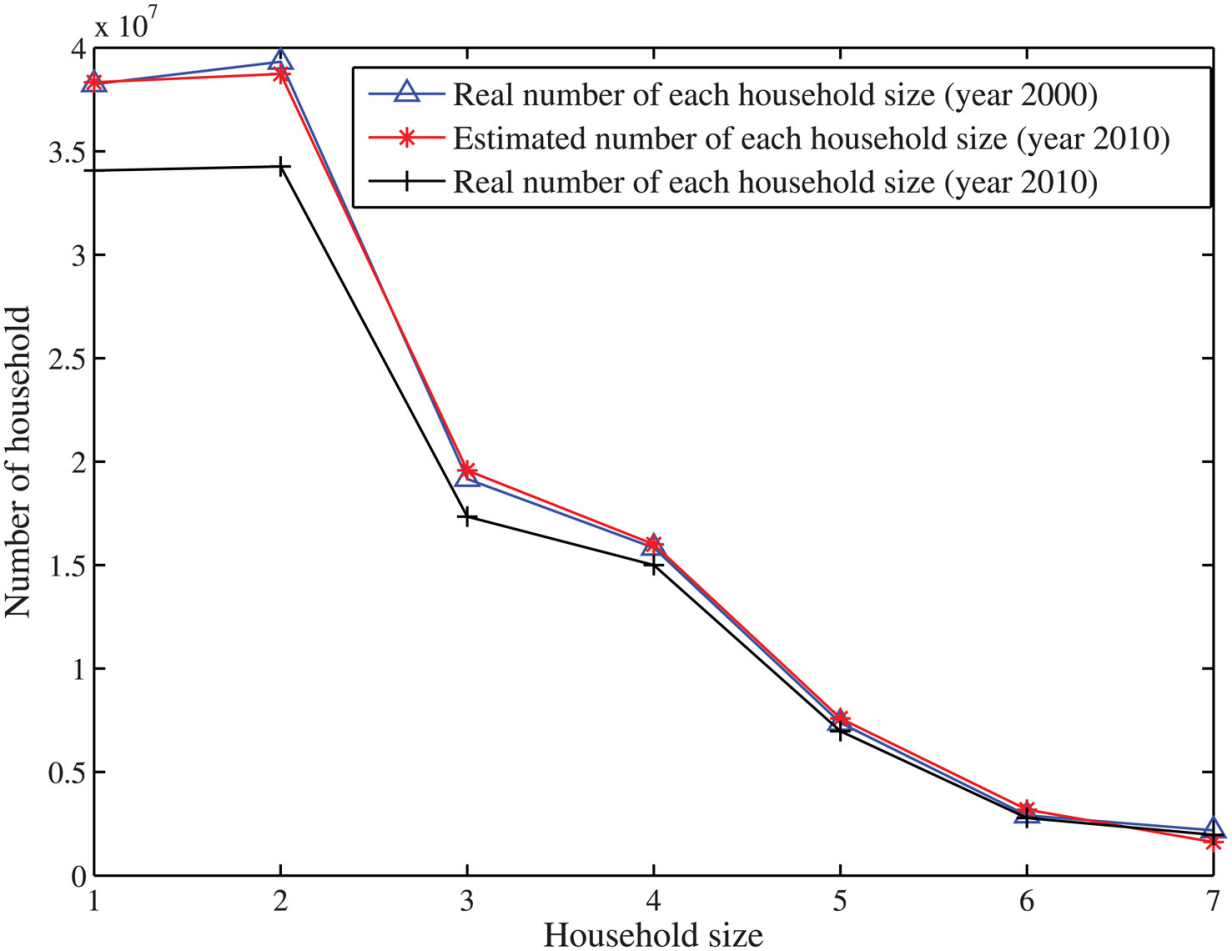
The prediction of number of household size distribution for US in the year 2010.

In stage 3, the numbers of each household size are estimated by solving the total household size Model (16). As seen in Fig 11, the difference between the estimation and the real values is quite close, which again shows the accuracy of our proposed method.

**Fig 12:**
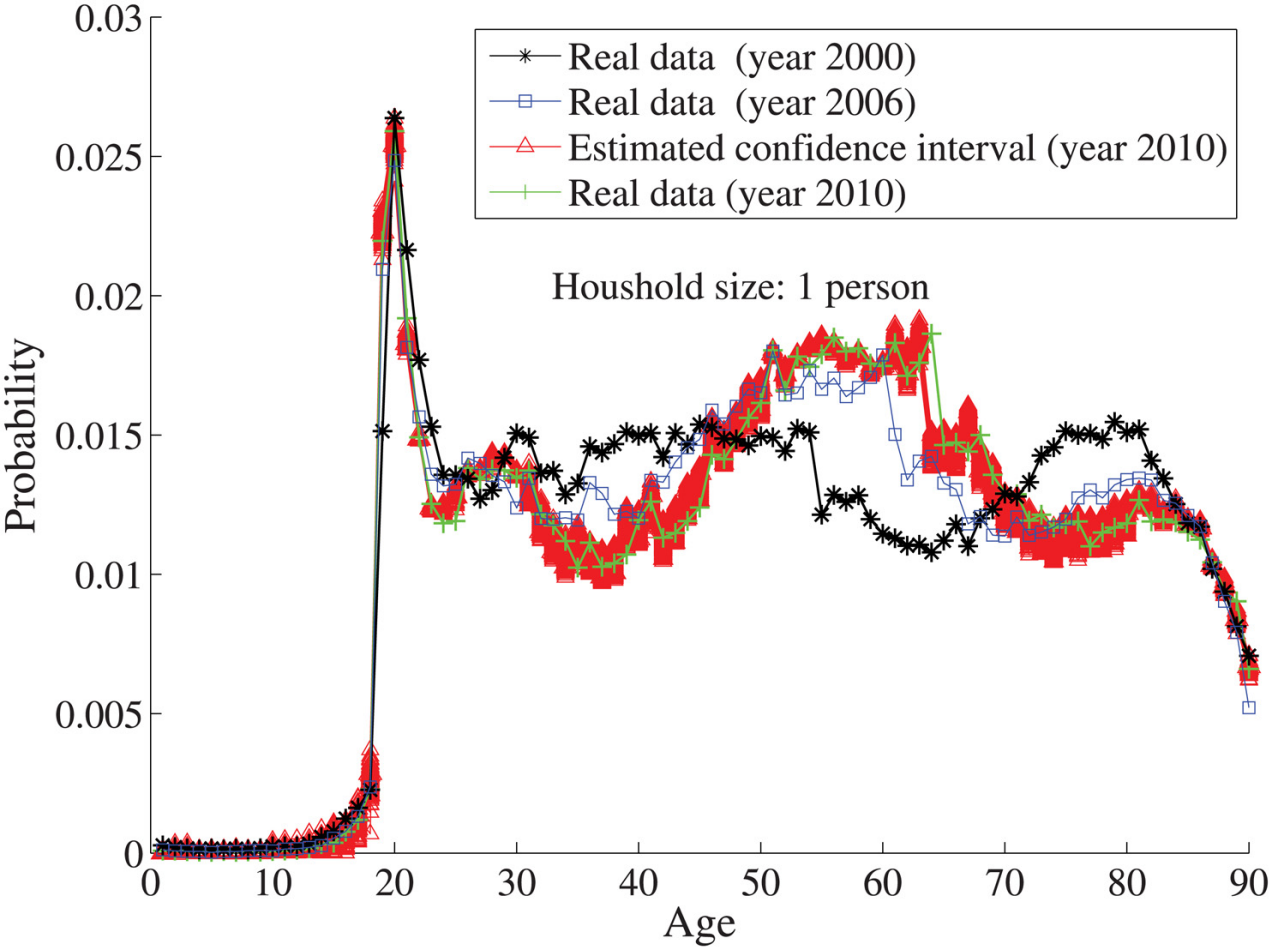
The illustration of the robustness of the estimation compared with their real values for household size 1.

In addition, we also look at the cases when the error term bound $\bar{\xi }\neq 0$. Let’s say, $\bar{\xi }=2$ implying that ${\bar{\xi }}_{k}\left(t+1\right)$ is randomly distributed in the interval [−2 2]. By repeating the simulations many times over (200 times in our case), we can verify the robustness of our estimations. We take the household size 1 as an example. As seen in Fig 12, most of the real values of the age distribution are located inside the red area generated by the estimations.

**Fig 13:**
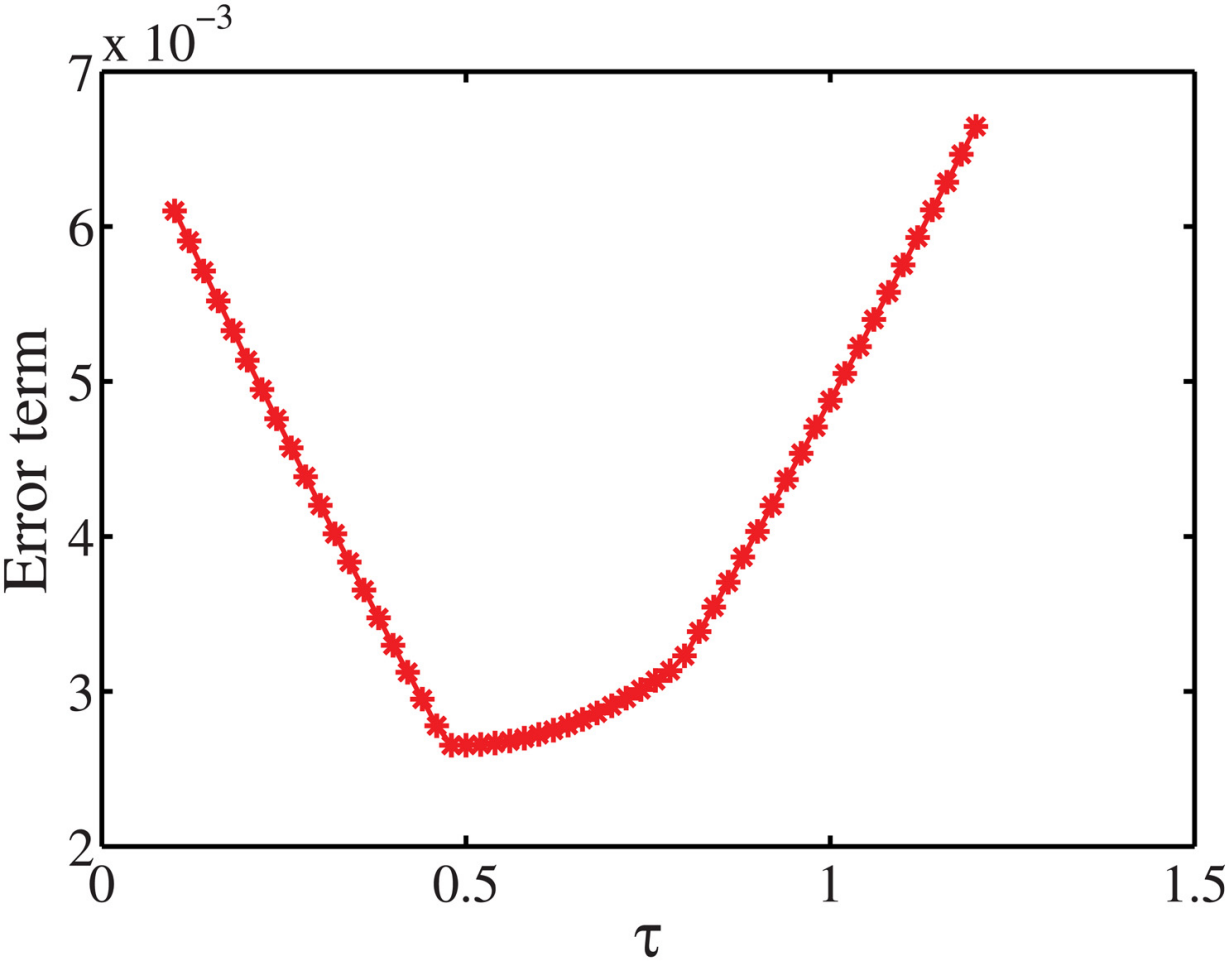
Error term with respect to the parameters *τ*.

**Fig 14:**
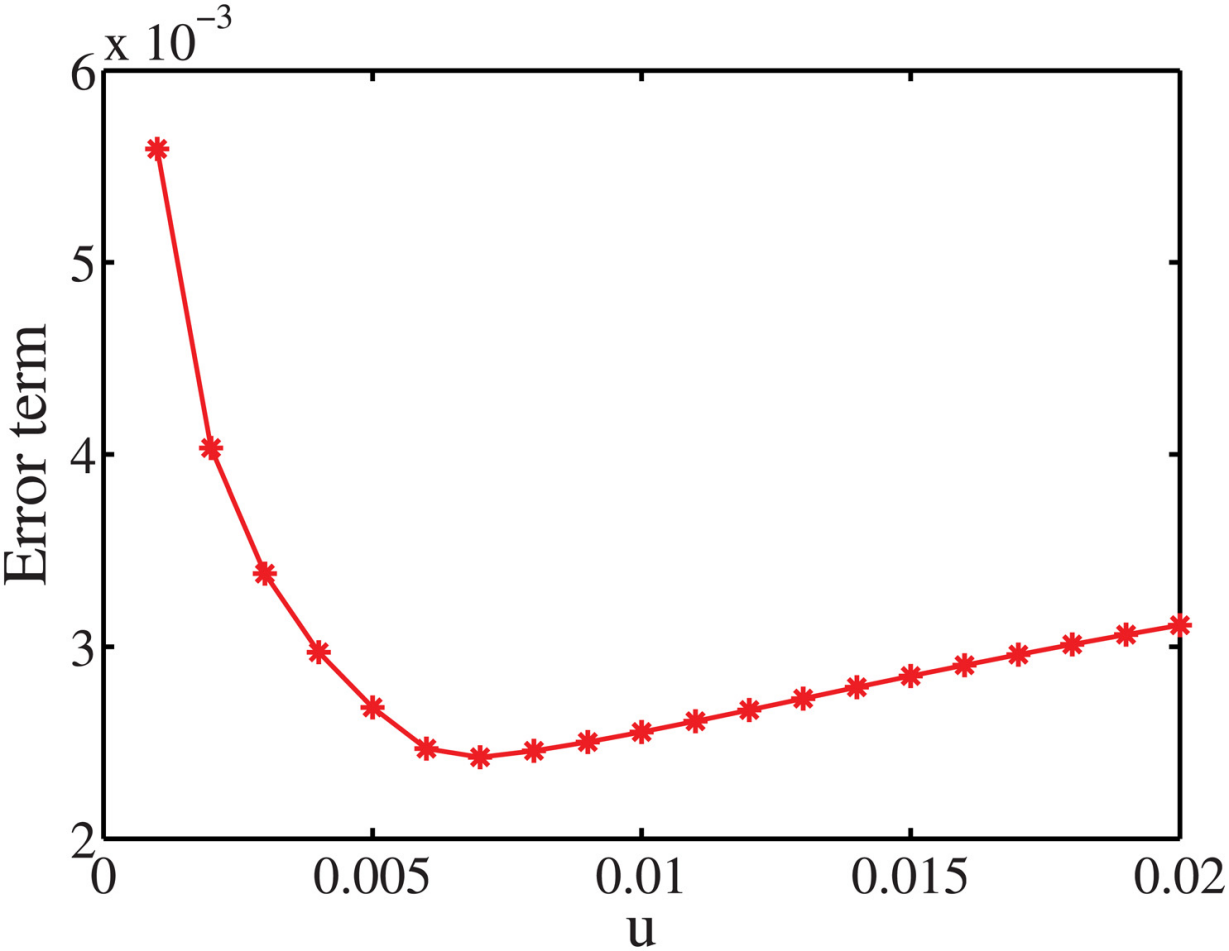
Error term with respect to the parameters *u*.

To investigate how the parameters *τ* and *u* affect the performance of the estimation, we set different values for them and see how the error term $Error\left(\hat{X}\left(t+1\right)\right)=\frac{||\hat{X}{\left(t+1\right)-X\left(t+1\right)||}_{1}}{{||X\left(t+1\right)||}_{1}}$ changes, where ‖.‖_1_ denotes the *L*_1_ norm. Fig 13 shows the error term against different values of *τ*. We observed that when *τ* is around 0.5, the proposed algorithm achieves its best performance. This is reasonable since the diagonal elements *W* ^*T*^ *W* is just the number of persons in each household size when *τ* = 0.5. On the other hand, Fig 14 shows how the parameter *u* impacts the estimation error. By setting different values for *u* in the interval [0 0.2], we observe that the performance of the algorithm provides the best fit when *u* ∈ [0.005 0.01].

## Discussion and Conclusions

In this paper, we have demonstrated a new method that estimates the development of age and household’s size distributions. The procedure consists of three models in three coupled stages, we referred to as: *the age-structured population model* in stage 1 where the age distribution of countries’ population was predicted; *the individual household size model* in stage 2 where the age distribution of each individual household size was estimated; and *the total household size model* in stage 3 where the number of different household sizes was derived by projecting the age distribution of total population onto the age distributions of individual household sizes. The procedure described here indicates that demographic trends can be accurately estimated using entropy as an optimisation variable, which we believe will be of potential interest to both academics and practitioners alike. We have illustrated and validated the correctness and accuracy of the proposed method using US data. While we have considered age and household size distributions in this article, we note that the method we have demonstrated is general and versatile enough to be extended to other time dependent demographic variables.
